# The Hidden Threat: Rodent-Borne Viruses and Their Impact on Public Health

**DOI:** 10.3390/v17060809

**Published:** 2025-06-02

**Authors:** Awad A. Shehata, Rokshana Parvin, Shadia Tasnim, Phelipe Magalhães Duarte, Alfonso J. Rodriguez-Morales, Shereen Basiouni

**Affiliations:** 1 TUM School of Natural Sciences, Bavarian NMR Center (BNMRZ), Structural Membrane Biochemistry, Technical University of Munich, 85748 Garching, Germany; 2 Department of Pathology, Faculty of Veterinary Science, Bangladesh Agricultural University, Mymensingh 2200, Bangladesh; rokshana.parvin@bau.edu.bd (R.P.); sadiatasnim562@gmail.com (S.T.); 3 Postgraduate Program in Animal Bioscience, Federal Rural University of Pernambuco (UFRPE), Recife, Pernambuco 52171-900, Brazil; duarte.phe@gmail.com; 4 Faculty of Health Sciences, Universidad Científica del Sur, Lima 15307, Peru; arodriguezmo@cientifica.edu.pe; 5 Grupo de Investigación Biomedicina, Faculty of Medicine, Fundación Universitaria Autónoma de las Américas-Institución Universitaria Visión de las Américas, Pereira 660003, Colombia; 6 Institute of Molecular Physiology, Johannes Gutenberg University, 55128 Mainz, Germany; sbasioun@uni-mainz.de

**Keywords:** Rodents, Arenaviruses, Hantaviruses, Coronaviruses, Picornaviruses, Poxviruses, zoonotic diseases

## Abstract

Rodents represent the most diverse order of mammals, comprising over 2200 species and nearly 42% of global mammalian biodiversity. They are major reservoirs of zoonotic pathogens, including viruses, bacteria, protozoa, and fungi, and are particularly effective at transmitting diseases, especially synanthropic species that live in close proximity to humans. As of April 2025, approximately 15,205 rodent-associated viruses have been identified across 32 viral families. Among these, key zoonotic agents belong to the *Arenaviridae*, *Hantaviridae*, *Picornaviridae*, *Coronaviridae*, and *Poxviridae* families. Due to their adaptability to both urban and rural environments, rodents serve as efficient vectors across diverse ecological landscapes. Environmental and anthropogenic factors, such as climate change, urbanization, deforestation, and emerging pathogens, are increasingly linked to rising outbreaks of rodent-borne diseases. This review synthesizes current knowledge on rodent-borne viral zoonoses, focusing on their taxonomy, biology, host associations, transmission dynamics, clinical impact, and public health significance. It underscores the critical need for early detection, effective surveillance, and integrated control strategies. A multidisciplinary approach, including enhanced vector control, improved environmental sanitation, and targeted public education, is essential for mitigating the growing threat of rodent-borne zoonoses to global health.

## 1. Introduction

Rodents have long posed a challenge for humans, as they damage crops, invade homes, and spread various diseases caused by several pathogens, including bacteria, protozoa, fungi, and viruses [1,2]. They are the largest group of living mammals, comprising approximately 2277 recognized species across 33 families, which constitute about 42% of global mammalian biodiversity [3]. While hundreds of rodent species are known to be reservoirs for several zoonotic pathogens, this represents only a fraction of the more than 2000 species in the *Rodentia* order [4]. Among them, there are species that are more often dangerous to humans in epidemiological terms [5,6].

The lifestyle of rodents significantly influences their ability to carry and transmit zoonotic pathogens. Sinanthropus rodents are much more likely to act as reservoirs for these pathogens compared to those that inhabit natural habitats [4]. This relationship has existed for at least 15,000 years, dating back to when mice coexisted with early human habitats in the Near East, a trend that intensified with the advent of agriculture [7].

Today, over 150 rodent species exhibit some degree of synanthropy, with six common species, including black rats (*Rattus rattus* Linnaeus, 1758), Norway rats (*Rattus norvegicus* Berkenhout, 1769), and house mice (*Mus musculus* Linnaeus, 1758), being almost entirely dependent on human environments. Each of these species is capable of carrying several zoonotic pathogens [4].

As of April 2025, the Database of Rodent-associated Viruses (DRodVir/ZOVER) has reported about 15,205 rodent-associated viruses from 32 families. The rodent families *Muridae*, *Cricetidae*, and *Sciuridae* account for the majority of species known to harbor these viruses. Rodents are acknowledged as reservoirs for nearly 60 zoonotic diseases [8] and play a critical role in the spread of these diseases through various mechanisms [1]. Rodent-associated diseases that impact public health include bacterial infections (*Mycobacterium* tuberculosis, *Mycobacterium microti*, *Escherichia coli*, *Francisella tularensis*, *Bartonella* spp., *Listeria monocytogenes*, *Salmonella* spp., *Leptospira* spp., *Borrelia burgdorferi*, *Coxiella burnetii*, and *Ehrlichia* spp.), viral infections (arenaviruses, hantaviruses, coronaviruses, paramyxoviruses, picornaviruses, and poxviruses), parasitic infections (*Leishmania* spp., *Toxoplasma gondii*, *Babesia microti*, and *Capillaria hepatica*), and fungal infections (*Trichophyton* spp. and *Histoplasma capsulatum*) [8,9]. The most commonly identified rodent-associated viruses are as follows: *Coronaviridae* (11,938 viruses; 21%), *Flaviviridae* (11,980 viruses; 21.1%), *Hantaviridae* (5222 viruses; 9.2%), *Paramyxoviridae* (2788 viruses; 4.9%), *Arenaviridae* (1576 viruses; 2.8%), *Hepeviridae* (1047 viruses; 1.8%), and *Poxviridae* (641 viruses; 1.1%) (DRodVir/ZOVER).

Rodents’ role as zoonotic reservoirs is closely linked to their high populations and frequent interactions with humans, which increase the risk of transmitting pathogens [10]. Synanthropic rodents also tend to have fluctuating population sizes, making them ideal hosts for diverse pathogens that can infect multiple species [4,10]. The frequency of rodent-borne diseases has been steadily rising in recent years due to several drivers, including the emergence of new pathogens, environmental shifts, climatic changes, and anthropogenic activities, such as urbanization, deforestation, and agricultural intensification. These drivers not only promote the expansion of rodent populations but also alter the dynamics of pathogen transmission, facilitating the spread of pathogens that have previously been limited to specific regions [11,12,13]. Furthermore, it was estimated that the global urban population may reach 2.1 billion by 2030, suggesting substantial ecological and health-related shifts, particularly concerning these rodents [14,15]. This review aims to shed light on the main viruses transmitted by rodents and to highlight the complex interplay between rodent ecology, human activity, and the emergence of zoonotic viral diseases. This work seeks to promote a proactive and multidisciplinary approach to disease prevention, emphasizing surveillance, rodent control, and community education as key components in effective risk mitigation.

This review was conducted through a comprehensive literature search and data synthesis to provide an updated overview of rodent-borne viruses and their epidemiological significance. Relevant scientific articles, databases, and reports were identified using electronic databases, including PubMed, Web of Science, Scopus, and Google Scholar. Search terms included combinations of keywords, such as “rodent-borne viruses”, “synanthropic rodents”, “zoonotic pathogens”, “rodent ecology”, and “disease emergence”. This review incorporated data from the Database of Rodent-associated Viruses (DRodVir/ZOVER), as of April 2025, to analyze the diversity and distribution of viruses linked to rodent hosts globally. Studies covering rodent ecology, human-rodent interactions, and the impact of anthropogenic and environmental changes on the dynamics of rodent-borne disease transmission were also included to explore the multifactorial drivers of zoonotic outbreaks. The selection criteria prioritized recent peer-reviewed articles, reviews, and authoritative reports published in English, with a focus on those providing epidemiological, ecological, and molecular insights. Older seminal works were included where necessary to provide historical context. Data were critically assessed and synthesized to highlight the links between rodent lifestyle (particularly synanthropy), virus diversity, and the risk of zoonotic diseases. The review aims to integrate multidisciplinary perspectives to support recommendations for surveillance, control strategies, and public health interventions.

## 2. Hantavirus

### 2.1. Characteristics of Hantaviruses and Their Molecular Relationships

The family *Hantaviridae*, which represents a serious public health concern, is divided into four subfamilies: *Actantavirinae*, *Mammantavirinae*, *Agantavirinae*, and *Repantavirinae*. Within the subfamily *Agantavirinae*, there are seven genera containing 53 species. The most significant genera are *Loanvirus*, *Mobatvirus*, and *Orthohantavirus* [16]. *Orthohantaviruses* are enveloped and appear spherical or oval at 80 to 120 nm in diameter. The genome consists of single-stranded, negative-sense RNA and is segmented (three segments): the L segment encodes RNA-dependent RNA polymerase (RdRp), while the M segment encodes a glycoprotein precursor (GPC), which subsequently splits into two proteins, Gn and Gc, and the S segment encodes the nucleocapsid protein [17,18].

**Figure 1:**
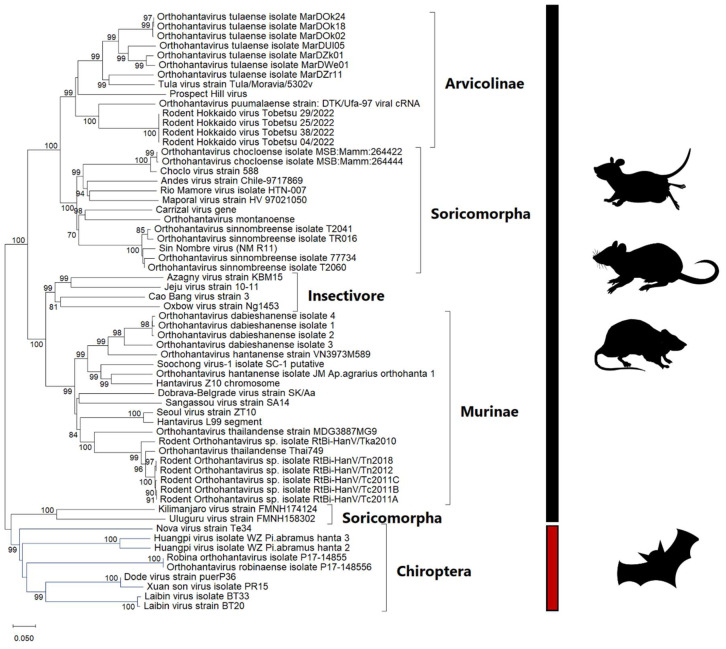
Phylogenetic tree of hantaviruses. The tree was constructed based on the segment L (RNA-dependent RNA polymerase gene) of 62 taxa. The maximum-likelihood tree was constructed using MEGAXI software with the GTR + G + I model. The bootstrap values shown are percentages of the 1000 replicates. The tree is scaled so that the branch lengths reflect the evolutionary distances used to build the phylogenetic tree.

**Figure 2:**
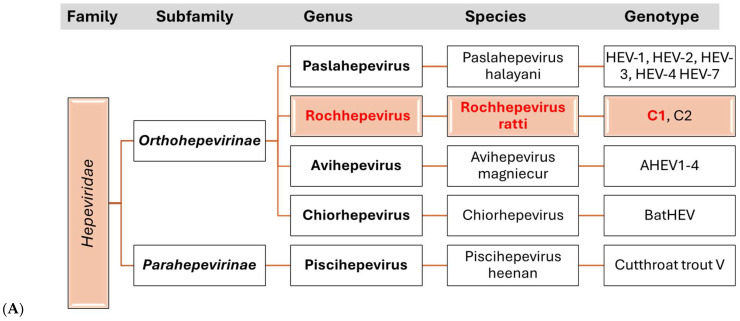

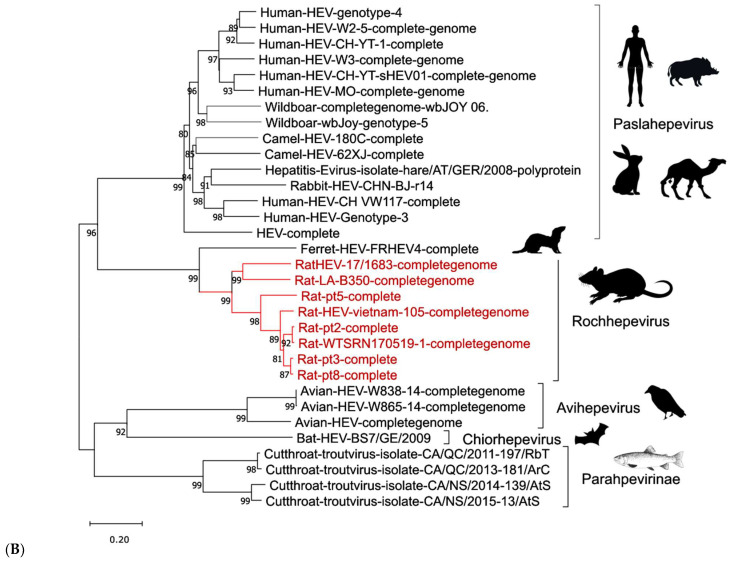
Taxonomy family *Hepeviridae* and Phylogeny. (**A**) The taxonomy includes reservoirs as follows: HEV-1 and HEV-2 = humans. HEV-3 and HEV-4 = house and wild swine, camel, and humans. EHV-7: humans and dromedary. C1: rodents and humans. C2: mink and ferrets. AHEV: chickens. BatHEV: bats. Cutthroat trout virus: trout. (**B**) Phylogenetic relationships of the viruses based on their taxonomy, with species-level clustering indicated at the end of each branch. Rat-originated *Rachhepevirus* taxa are highlighted in red and are grouped into two distinct clusters, C1 and C2. The tree was constructed using the complete genome sequences of 32 taxa. The evolutionary distances were computed using the Maximum Composite Likelihood method and are expressed as the number of base substitutions per site. The phylogenetic tree is drawn to scale, with the branch lengths proportional to the evolutionary distances used in the analysis.

A phylogenetic reconstruction of hantaviruses using representative hantaviruses from various countries is shown in Figure 1. Viral genome sequences were sourced from the GISAID and NCBI databases. Multiple sequence alignment was performed with the MAFFT web tool [19], and a phylogenetic tree was constructed based on the maximum-likelihood tree, using MEGA XI software with the GTR + G + I model. The branch lengths were measured in terms of substitutions per site, and bootstrap values were calculated using 1000 replicates. It was found that hantaviruses are linked with various rodent subfamilies, as shown in Figure 2, and each form distinct phylogenetic branches.

### 2.2. Epidemiology

Although orthohantaviruses primarily infect small rodents, the first hantavirus, known as Thothrappalayam, was isolated from the Indian Asian house shrew (*Suncus murinus* Linnaeus, 1766) in 1964 [20]. Hantaviruses have also been identified in shrews (family *Soricidae*), moles (family *Talpidae*), and bats (order *Chiroptera*). These hosts are typically asymptomatic carriers, continuously shedding the virus into the environment through their excretions, such as urine, feces, and saliva. This shedding increases the risk of human exposure and infection [21].

Hantaviruses are categorized based on their geographical distribution into “Old World” and “New World” types. Old-world hantaviruses, including Puumala virus [transmitted by Bank vole (*Myodes glareolus* Schreber, 1780)] and Dobrava–Belgrade virus [transmitted by Yellow-necked mice (*Apodemus flavicollis*)], are primarily prevalent in Europe and Asia [22,23,24]. However, the Hantaan, Amur, and Soochong viruses are distributed in Russia, China, and South Korea [25,26,27].

Hantaviruses are strongly associated with two serious diseases in humans: Hemorrhagic Fever with Renal Syndrome (HFRS; caused by “Old World” viruses) and Hantavirus Pulmonary Syndrome (HPS; caused by “New World” hantaviruses), also known as Hantavirus Cardiopulmonary Syndrome (HCPS) [24,28].

Notably, the distribution of hantavirus species is closely linked to their rodent hosts. In Eurasia, HFRS is caused by several viruses, including the Hantaan virus, Dobrava–Belgrade virus, Seoul virus, Puumala virus, and Tula virus [24,28]. However, HPS, or HCPS, is a severe respiratory illness primarily reported in the Americas. While these viruses remain asymptomatic in their rodent hosts, they can be lethal to humans. The primary transmission route is through the inhalation of aerosolized viral particles; however, rare cases of direct transmission via rodent bites have also been reported. Human-to-human transmission of the Andes virus is exceedingly rare and has only been documented in cases in Argentina and Chile [29,30,31].

The incidence of HFRS follows cyclical patterns influenced by the dynamics of rodent populations, which fluctuate in response to seasonal and climatic changes. The first outbreak of HFRS occurred between 1950 and 1953, during which over 3000 U.S. soldiers were infected during the Korean War [32]. This event highlights how human activities can contribute to the emergence and spread of new infectious diseases. Surges in rodent populations typically happen every three to four years and correlate with increased human infections. These peaks usually occur in the summer and autumn when outdoor exposure is high, as well as in autumn and winter when rodents seek shelter indoors, raising the risk of transmission [33]. Globally, hantaviruses are estimated to infect more than 150,000 individuals annually [34]. It should be emphasized that each orthohantavirus species has a specific host (see Table 1).

### 2.3. Clinical Picture

HFRS begins with flu-like symptoms but can escalate to shock, renal failure, and hemorrhagic complications. In contrast, HCPS causes fever and can lead to severe respiratory distress due to lung edema, often resulting in fatal outcomes [35]. Of note, HFRS caused by the Hantaan, Amur, and Dobrava viruses tends to be more severe, with a mortality rate ranging from 5 to 15%. In contrast, the Seoul virus causes moderate illness, while the Puumala and Saaremaa viruses lead to milder forms of the disease, with mortality rates of less than 1% [24].

The severity of HFRS varies based on the virus strain. Puumala virus infections often result in nephropathia epidemica, a mild form of HFRS, with a case fatality rate of 0.08% to 0.4%. In contrast, Dobrava infections can be much more severe, with fatality rates ranging from 9% to 12% [36,37]. In 2012, over 2800 cases of infection linked to the Puumala virus were reported. The Hantaan virus, primarily infecting *Apodemus agrarius* (Pallas, 1771), remains confined mainly to Asia [23,38,39]. The Seoul virus has a worldwide distribution, carried by *Rattus norvegicus* (Berkenhout, 1769) and *Rattus rattus*, which thrive along human transportation routes. The Seoul virus generally has a moderate fatality rate of around 1%, whereas HNTV is the most lethal, causing fatalities in up to 15% of cases, particularly in Asia and Far East Russia [23,40]

### 2.4. Diagnosis and Control

The global dynamics of hantavirus infections are closely tied to the availability and application of accurate diagnostic tools, which vary significantly across regions. Traditionally, serological assays, such as ELISA and immunofluorescence, remain the primary methods for hantavirus diagnosis worldwide due to their relative simplicity and accessibility, especially in resource-limited settings [41]. ELISA, a gold standard test, can be used for the detection of IgM and IgG antibodies against orthohantavirus antigens. However, these methods have limitations, including cross-reactivity and delayed antibody response, which can hinder early detection and timely intervention. Molecular methods, such as RT-PCR, enable rapid identification of viral RNA. However, it is less commonly used due to the transient nature of viremia [24,42].

Currently, there is no approved vaccine or effective treatment for hantavirus infections. As a result, raising public awareness and implementing preventive measures are the primary strategies for reducing the risk of hantaviruses [24]. Combining serologic tests, such as IgM ELISA, with RT-PCR provides a highly sensitive and effective approach for laboratory diagnosis [43]. Moreover, advanced high-throughput platforms, including microarrays and next-generation sequencing (NGS), offer valuable tools for improving viral detection and genomic profiling. These technologies support the discovery of new hantavirus variants and enable surveillance of viral mutations. In particular, the use of MinION nanopore sequencing, a palm-sized portable sequencer that is smaller and cheaper than conventional sequencing platforms, in field settings shows potential for the rapid analysis of the Hantaan virus from both clinical and animal samples during outbreaks [44]. This portable sequencing method could significantly contribute to genome-based diagnostics and targeted control strategies in regions where HFRS is highly prevalent.

To fully harness the epidemiological benefits of these diagnostic advancements, it is essential to strengthen diagnostic capacity globally and ensure equitable access to such technologies. Continued investment in public health infrastructure, workforce training, and cross-sector collaboration will be vital to effectively respond to the evolving hantavirus threat in a changing global environment.

## 3. Hepatitis E Virus

### 3.1. Etiology

Hepatitis E virus (HEV), also known as *Paslahepevirus balayani*, is a significant pathogen that primarily causes acute or chronic liver inflammation in humans. The World Health Organization (WHO) estimates that approximately 20 million people are infected with HEV each year, resulting in around 3.3 million symptomatic cases worldwide [45]. Although HEV is now recognized as a major cause of liver disease, it was only identified as a distinct virus in the early 1980s [46]. Before that, HEV outbreaks were often mistakenly attributed to the hepatitis A virus (HAV) due to their similar clinical and epidemiological characteristics [46].

In 1983, Soviet-Russian researcher Mikhail Balayan conducted a self-experiment using a stool sample from an infected hepatitis patient. He discovered novel virus particles in his stool that did not react with HAV [47]. HEV was definitively characterized through genome sequencing in 1991 [48,49]. While the focus was initially on human infections, novel HEV strains were first identified in pigs in 1997 and were later classified as genotype 3. Additional HEV genotypes have been found in pigs and humans in Southeast Asia, as well as in dromedaries and humans in the Middle East, highlighting the zoonotic potential of the virus [50,51].

HEV is a single-stranded RNA virus with an icosahedral capsid of around 30 nm in diameter. The viral genome is approximately 7000 nucleotides in length and contains three open reading frames (ORFs): (i) ORF1 encodes a non-structural protein with RNA-dependent RNA polymerase activity, (ii) ORF2 encodes the capsid protein, and (iii) ORF3 encodes a phosphoprotein involved in the release of viral particles [52]. HEV belongs to the *Hepeviridae* family, comprising several species, genotypes, and genera (Figure 2). Human-pathogenic genotypes 1–4 and 7 are classified under *Paslahepevirus balayani*, while rat HEV belongs to *Rocahepevirus ratti* [53]. Rat HEV was first postulated based on serological findings indicating the presence of HEV-reactive antibodies in rodents. However, it was not until 2010 that the virus was identified in wild rats in Hamburg, Germany [54]. Further research on the virus revealed it to be genetically distinct from previously identified HEV strains, with the rat HEV genome containing an additional ORF4 not found in other strains [55].

Genotypes 1 and 2 primarily infect humans and can be transmitted through contaminated water, especially in regions with poor sanitation. Pigs and wild boars can transmit genotypes 3 and 4; however, other animals may also harbor the virus. These strains are typically transmitted to humans via undercooked meat products. Genotype 3 is prevalent worldwide, while genotype 4 is predominantly found in Southeast Asia. Genotype 7, identified in dromedaries and a human case in the Middle East, remains poorly studied [51]. In Germany, genotype 3 infections are most commonly associated with pigs and wild boars [52]. The number of reported human cases rose from around 100 in 2001 to over 3000 annually by 2019, with figures consistently remaining high, ranging from 3000 to 4500 cases [56].

### 3.2. Epidemiology

In 2010, HEV-like viruses were detected in rats in Germany [54]. These strains were distinct from the known HEV types and were later classified as *Rocahepevirus ratti* species [53]. Initially, only rats were thought to be infected, but subsequent studies revealed evidence of rat HEV antibodies in humans, suggesting the possibility of human infections. In 2018, the first human case of rat HEV infection was confirmed in Hong Kong, suggesting a new zoonotic transmission route of HEV [57]. Further cases have since been documented in Canada, Spain, France, and Hong Kong [56,57,58]. Phylogenetic analyses have confirmed that rat HEV is a separate virus species, classified under *Rocahepevirus ratti* within the genus *Rocahepevirus* [53]. The global spread of rat HEV has been confirmed in the USA, China, and Indonesia [59,60,61]. In Germany, rat HEV has been detected in wild brown rats in several regions, with detection rates varying from 2.7% to 35.3% [62]. There is no consensus on whether rats are the primary reservoir for rat HEV or if other animal species can transmit the virus.

### 3.3. Clinical Picture

HEV infections in humans can cause a range of clinical signs. Acute hepatitis, characterized by symptoms such as malaise, fever, jaundice, and abdominal pain, is the most common manifestation. The disease usually resolves on its own in healthy individuals, but it can lead to severe manifestations in persons with pre-existing liver conditions or for pregnant women infected with genotype 1 [63]. Immunocompromised individuals, particularly transplant recipients, may develop chronic HEV infections that persist for months or even years, potentially leading to life-threatening cirrhosis. Extrahepatic manifestations, including Guillain–Barré syndrome and neuralgic amyotrophy, have also been reported, though the exact relationship between HEV and these conditions remains unclear [64].

### 3.4. Diagnosis and Control

Acute HEV infection is commonly identified serologically through the detection of IgM and IgG. However, the reliability of these serological tests varies significantly depending on the commercial assay used [65,66]. The detection of RNA using RT-PCR in samples, including stool, serum, or liver tissue, is also utilized for diagnostic purposes. Diagnostic challenges persist in immunocompromised individuals and organ transplant recipients, where the typical antibody response may be delayed or absent [67].

Currently, no targeted treatment can modify the progression of acute HEV. Since the infection is typically self-limiting, most cases do not require hospitalization. However, it is crucial to avoid the use of medications that may impair liver function, such as acetaminophen or paracetamol. Hospital admission becomes necessary in cases of fulminant hepatitis and should also be considered for pregnant women exhibiting symptoms. For immunocompromised individuals with chronic HEV, antiviral therapy with ribavirin has shown clinical benefits. In select cases, interferon has also been used successfully as a treatment option [45].

Prevention is the most effective strategy against HEV. At the population level, reducing transmission primarily involves maintaining safe public water supplies and ensuring efficient sanitation systems for human waste disposal, particularly in endemic regions such as South Asia, parts of Africa, and areas affected by humanitarian crises [68]. Emergency water, sanitation, and hygiene interventions are especially critical in refugee camps and displaced communities, where outbreaks are more frequent. On an individual level, the risk of infection can be minimized by practicing good personal hygiene and avoiding the consumption of water or ice from unknown or potentially unsafe sources [45].

HEV prevention should be part of water safety, disaster preparedness, and maternal health plans, combined with rodent control, to effectively reduce transmission worldwide. Addressing the ongoing challenges of HEV and its zoonotic potential linked to rodents requires a comprehensive and integrated strategy. (i) National health systems in endemic countries should integrate HEV and rodent surveillance within public health frameworks, alongside investments in water, sanitation, and rodent control. (ii) In crisis areas, such as South Sudan, Gaza, Yemen, and Ukraine, rapid water, sanitation, and hygiene interventions and rodent management efforts paired with community education are essential. (iii) Global agencies should accelerate vaccine development and equitable distribution. Furthermore, cross-sectoral collaboration under the One Health framework is critical to understanding the ecological dynamics of rodent-borne HEV strains and mitigating the emergence of new zoonotic threats.

## 4. Arenaviruses

### 4.1. Etiology

Arenaviruses, belonging to the Family *Arenaviridae*, are single-stranded, negative-sense RNA viruses [60,69]. The genome consists of two segments: an L segment that encodes the Z protein and the L protein, which serves as an RNA-dependent RNA polymerase, and an S segment that encodes the nucleoprotein (NP) and the glycoprotein precursor (GPC) [70,71].

During infection, the NP and L proteins are synthesized first, followed by the GPC and Z proteins, ensuring a controlled sequence of viral protein expression [72]. The L protein is essential for viral RNA synthesis and is a component of ribonucleoprotein (RNP) complexes [73,74]. Meanwhile, the matrix protein Z contributes to viral assembly and budding and can inhibit RNA synthesis at higher concentrations [75]. Additionally, NP and Z are involved in modulating the host’s interferon response [76]. The GPC undergoes post-translational cleavage into GP1 and GP2, with the stable signal peptide (SPP) assisting in maturation and trafficking [77,78]. GP1 facilitates viral entry by binding to host receptors, such as α-dystroglycan [79], while GP2 is crucial for viral envelope fusion [80].

**Figure 3:**
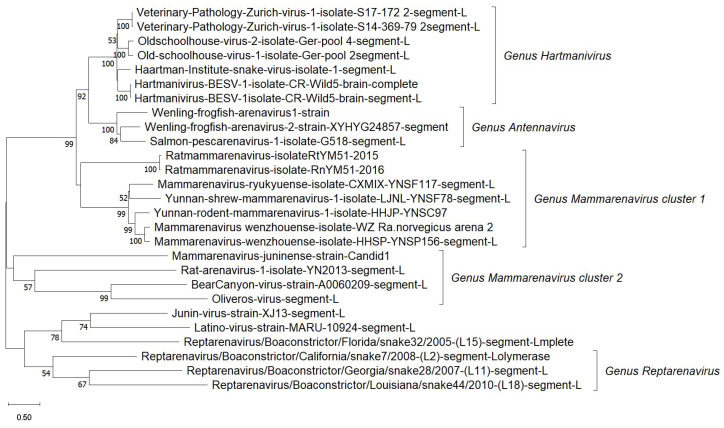
Phylogenetic relationships of arenaviruses. The tree was constructed using the complete L-segment genome of 27 taxa. The L segment sequences from four genera—*Reptarenavirus*, *Hartmanivirus*, *Antennavirus*, and *Mammarenavirus*—form distinct clusters. Notably, *Mammarenavirus* is further divided into two separate clusters based on the L gene, indicating significant genetic variation within the genus. The evolutionary distances were computed using the Maximum Composite Likelihood method and are in the units of the number of base substitutions per site.

The Arenaviridae family consists of five genera: Mammarenavirus, which mainly infects mammals, especially rodents, and Innmovirus [81]. Arenaviruses generally exhibit strong host specificity, often restricted to a single rodent species or even subspecies, suggesting a high degree of co-evolution and adaptation [82,83]. Among these, the Arenaviridae family, specifically the Mammarenavirus genus, includes the majority of arenaviruses known to cause zoonotic diseases. In contrast, Antennavirus and Hartmanivirus are more genetically distinct from the other genera (Figure 3), and their natural hosts remain largely unknown. Certain arenaviruses pose serious health risks to humans. For instance, the Lassa virus, which causes Lassa fever, is estimated to infect approximately 200,000 people annually in West Africa. Another example is lymphocytic choriomeningitis virus (LCMV), which is commonly transmitted through exposure to urine, droppings, or saliva from infected rodents, particularly house mice (M. musculus) and Syrian golden hamsters (Mesocricetus auratus Waterhouse, 1839) [84].

### 4.2. Epidemiology

The Lassa virus was first identified in 1969 in Lassa, Nigeria. Since its discovery, infections have also been documented in several countries outside of Africa, including the United States, Japan, and Europe [85]. Transmission can occur via inhalation of aerosolized particles, mucosal contact, or the ingestion of contaminated food [86]. Vertical transmission in rodents can lead to persistent infections, allowing lifelong viral shedding [87]. Experimental studies have demonstrated that infected male rodents produce fewer scent proteins, which are critical for attracting mates and signaling reproductive fitness [69]. This reduction in scent marking may lead to decreased mating success, suggesting that infected males could be less likely to reproduce and, consequently, might contribute less to the transmission of viruses, such as LCMV, within rodent populations. However, direct evidence linking reduced scent protein production to lower mating frequency and decreased transmission rates in natural settings remains limited and requires further investigation. House mice are the primary reservoirs for LCMV, but pet hamsters have also been implicated in human infections [88,89]. Although experimental studies have identified mosquitoes and ticks as potential vectors for LCMV transmission, their role in natural transmission cycles is still unclear [79]. Understanding the impact of altered mating behavior on virus spread could be important for modeling LCMV dynamics and targeting control measures [89].

In primates, transmission primarily occurs through the oral ingestion of contaminated food, with initial viral replication occurring in the gastric mucosa before systemic spread [90]. Captive primates appear to be more susceptible than wild populations, with infections often originating from facilities that have experienced prior outbreaks [91].

Lassa fever remains endemic in West Africa due to several factors that contribute to the spread of the virus. (i) Food scarcity during the dry season, which forces rodents to move closer to human settlements, increasing the risk of human exposure [92]. (ii) Biodiversity loss, particularly in Nigeria’s degraded forests and clearings, facilitates zoonotic disease spillover [93]. (iii) The consumption of rodents in Guinea has been linked to higher rates of Lassa virus infection among individuals who engage in this practice [94]. These elements collectively underscore the complex dynamics influencing Lassa fever’s distribution in West Africa.

LCMV is globally widespread due to the extensive presence of its rodent hosts [95]. Although the prevalence of infection is typically low, serological evidence suggests that prior exposure has occurred in humans and mammals across various continents [96,97]. The detection of LCMV-reactive antibodies in mammals across Europe, Asia, and the Americas implies broader exposure [98,99]. Outbreaks of LCMV are often linked to laboratory exposure, pet rodents, and rodent breeding facilities. Between 1971 and 1973, 48 infections were documented at the University of Rochester due to contaminated tumor cell lines [100]. A 2012 outbreak in Indiana, USA, was traced to rodent breeding facilities, with up to 47% of workers testing seropositive [101]. Additionally, pet hamsters have been associated with multiple outbreaks, including a significant one from 1973 to 1974 that affected 181 individuals across 12 states [102]. LCMV has also been transmitted through solid organ transplants, leading to severe cases in both the USA [103,104] and Australia [71]. Despite the associated risks, routine screening for LCMV in transplant donors remains uncommon [103]. However, increases in rodent populations, such as the recent “mouse plagues” in Australia, have been linked to a heightened risk of zoonotic transmission. During the 2020–2021 mouse plague in New South Wales and Queensland, several human cases of LCMV infection were documented, including a cluster of eight cases connected to extensive mouse exposure. These represent the first known human LCMV infections in Australia, emphasizing the need for increased awareness and routine screening in regions experiencing rodent population surges [105].

### 4.3. Clinical Picture

LCMV is usually associated with mild flu-like symptoms in humans. However, severe complications can arise, such as encephalitis and meningitis, particularly in immunocompromised individuals and organ transplant recipients [106]. Congenital LCMV infection during pregnancy can lead to fetal mortality or serious congenital disabilities, which may include microcephaly, hydrocephalus, and blindness [107]. Additionally, there is speculation that LCMV may play a role in the onset of multiple sclerosis [108]. In New World primates, LCMV infection can cause Callitrichid Hepatitis, a frequently fatal disease characterized by lesions in the liver, brain, and lymphoid tissues [109]. Infected primates exhibit symptoms such as jaundice, difficulty breathing, hemorrhage, and pleuropericardial effusion, with mortality typically occurring within 7 to 12 days [110].

Effective control measures are crucial in reducing the likelihood of future outbreaks. These measures include enhanced surveillance, improved diagnostic screening, and increased public awareness. Given the potential risks from laboratory exposure, pet rodents, and organ transplantation, it is essential to implement proactive strategies to prevent transmission and protect both human and animal health.

## 5. Ljungan Virus

Picornaviruses are a widely distributed family of viruses that infect a diverse range of hosts, including humans, mammals, birds, amphibians [111], and fish [112,113]. These viruses are known to cause diseases that affect multiple organ systems. One particular picornavirus, Ljungan virus (LV; Parechovirus B), has garnered scientific interest due to its potential association with human gestational disorders and type 1 diabetes [114,115]. LV was first identified in bank voles (*Myodes glareolus* Schreber, 1780) and was initially hypothesized to be a rodent-borne zoonotic virus [116]. LV RNA has since been detected in 12 species of voles, lemmings, mice, shrews, and squirrels in nine European countries, with an estimated mean prevalence of 15.2% [117].

**Figure 4:**
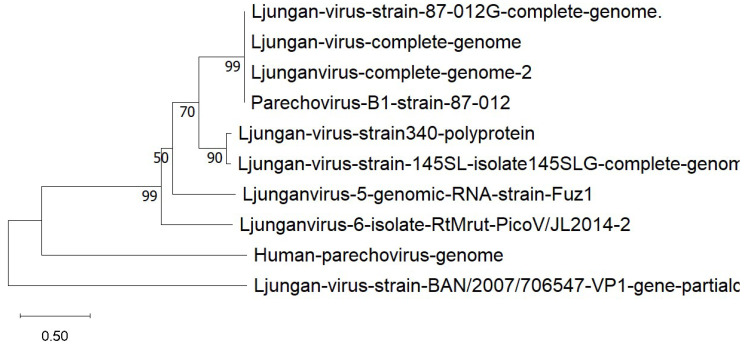
Phylogenetic relationships of the Ljungan virus. The tree was constructed based on the complete genome sequences of nine taxa and one partial VP1 sequence. The different genotypes are represented on separate branches. The evolutionary distances were computed using the Maximum Composite Likelihood method and are in the units of the number of base substitutions per site.

LV has a positive-sense, single-stranded RNA genome that encodes a single polyprotein, which is then cleaved into 11 functional proteins. The VP1 region is commonly used for genotypic classification [118,119]. To date, eight complete LV genomes have been sequenced, corresponding to five distinct genotypes [118,119,120]. Additionally, a potential sixth genotype (RtMrut-PicoV/JL2014-2) has been proposed as a new candidate member of the Parechovirus genus based on sequencing data from the northern red-backed vole (*Myodes rutilus* Pallas, 1779) in China [121], as shown in Figure 4.

Seroprevalence studies indicate a peak in LV-reactive antibodies among children, suggesting the possibility of a human-specific virus resembling LV rather than accurate zoonotic transmission [114]. This raises questions about the origin of LV-like infections in humans and highlights the need for further investigation into their epidemiology and potential reservoirs.

LV has been linked to myocarditis and type 1 diabetes in captive wild voles, particularly under stress conditions, as well as to gestational abnormalities in laboratory mice [122,123]. However, its impact on natural rodent population dynamics appears to be minimal [124]. While LV’s pathogenic role in humans remains unclear, its potential association with pregnancy complications and autoimmune diseases necessitates further research to clarify its clinical significance.

To summarize, Ljungan virus represents a complex and evolving topic within the Picornaviridae family. Although initially considered a rodent-borne zoonotic virus, the lack of confirmed human transmission and the presence of LV-reactive antibodies in children suggest alternative hypotheses regarding its epidemiology. Further genomic, serological, and epidemiological studies are necessary to determine the true host range, pathogenic potential, and relevance of LV to human health. Understanding the genetic diversity and transmission mechanisms of LV will be crucial in assessing its public health significance and potential role in human disease.

## 6. Poxviruses

Poxviruses, part of the *Poxviridae* family, are double-stranded DNA viruses measuring 200–400 nm in size, with a genome spanning 150–250 kbp [125]. These viruses have long affected humans and animals, with evidence of pox-like scars found on the Egyptian King Ramesses V (1100–1580 B.C.), who is believed to have died from smallpox. The earliest documented case of smallpox dates back to 4th-century Chinese texts [119]. Poxviruses can infect a broad range of hosts, including mammals, birds, reptiles, and insects, though some have more restricted host ranges due to gene-specific determinants that influence host susceptibility [126]. The *Poxviridae* family is divided into two subfamilies: *Chordopoxvirinae*, which infects vertebrates, and *Entomopoxvirinae*, which infects invertebrates. The *Chordopoxvirinae* subfamily consists of 18 genera, including *Orthopoxvirus*, *Parapoxvirus*, and *Yatapoxvirus*, which are known to contain zoonotic members [127,128].

Poxviruses typically enter the host through the skin, though orthopoxviruses can also invade via respiratory and mucosal routes. They primarily cause localized lesions that progress through the macular, papular, vesicular, and pustular stages. Some cases lead to systemic infections via viremia [129]. Rodents serve as significant reservoirs and vectors in the transmission of various poxviruses, including the Monkeypox Virus (Mpox) (Eslamkhah et al., 2025), cowpox virus (CPXV), and Squirrelpox. In this section, we will provide an overview of CPXV and Mpox.

### 6.1. Monkeypox Virus (MPXV)

Mpox was first identified in 1958 when it was isolated from monkeys in a Danish laboratory [130]. The first human case was recorded in 1970 in the Democratic Republic of the Congo [131]. Since then, cases have been reported in 11 African countries, including Nigeria, Cameroon, and the Central African Republic [132,133]. The first outbreak outside Africa occurred in the United States in 2003 due to infected prairie dogs that had been housed with rodents imported from Ghana [134]. Subsequent outbreaks have been recorded in Israel, the UK, Singapore, and the USA [135].

Mpox symptoms resemble those of smallpox but tend to be less severe [135]. The incubation period ranges from 6 to 16 days, with some cases extending up to three weeks. Initial symptoms include fever, swollen lymph nodes, severe headaches, muscle aches, and back pain. Within 1–3 days post-fever, maculopapular rashes appear, progressing through vesicular and pustular stages before crusting over in approximately 10 days [136].

The Mpox genome ranges from 185,309 bp (*GenBank*: MT903341.1, Mpox-M5320_M15_Bayelsa strain) [137] to 206,372 bp (*GenBank*: KC257459.1, Sudan 2005_01 strain) [138]. The recently identified Mpox strain MA001 has a genome of 197,124 bp, with over 200 putative open reading frames (ORFs). ORFs longer than 150 bp are considered functionally relevant. The MA001 strain retains all essential genes for *Orthopoxvirus* replication and includes four unique ORFs (*ORF 16*, *ORF 26*, *ORF 146*, and *ORF 179*) that may contribute to Mpox’s transmission and recent outbreaks. Further research is needed to determine their roles. Mpox, like most poxviruses, possesses inverted terminal repeat (ITR) sequences at both termini of its genome. These sequences contain A-T-rich hairpin loops that connect Mpox DNA strands [139].

Mpox spreads through direct or indirect contact with infected animals or humans. Zoonotic transmission occurs via bites, scratches, handling of infected animal products, or consuming undercooked meat [140,141]. Human-to-human transmission primarily occurs through respiratory droplets, skin lesions, or contaminated materials [139]. Sexual transmission has also been documented, particularly among men who have sex with men [135]. The virus enters through broken skin, mucous membranes, or the respiratory tract, with an incubation period ranging from 5 to 21 days. Further research is necessary to understand Mpox transmission, reservoir hosts, and the genetic adaptations that contribute to its spread. Mpox consists of two genetic clades: (i) the West African clade, primarily found from western Cameroon to Sierra Leone. (ii) The Congo Basin clade, circulating from central to southern Cameroon and the Republic of the Congo [138,142].

Mortality rates differ between these clades, with the Congo Basin strain exhibiting a 10.6% fatality rate, compared to 3.6% for the West African strain. The age profile of the infection has shifted from young children (with an average age of 4 years in the 1970s) to young adults (with an average age of 21 years in 2010–2019) [143].

Comparable to human smallpox, the incubation period for Mpox in humans is 10 to 14 days. The clinical presentation of Mpox also closely resembles that of human smallpox. Infected individuals are contagious during the first week after the appearance of the erythema. Similar to human smallpox, a prodromal phase characterized by fever and malaise typically occurs approximately two days before the onset of the rash. However, unlike human smallpox, the majority of patients with an MPXV infection exhibit pronounced unilateral or bilateral lymphadenopathy, which can affect various lymph nodes. The disease progression follows a similar pattern to human smallpox, characterized by the spreading of erythema in a centrifugal manner. To date, no hemorrhagic courses have been observed in monkeypox cases [144].

### 6.2. Cowpox Virus (CPXV)

Human infections with cowpox virus (CPXV) are rare but can occur, primarily in individuals who have direct contact with infected animals, such as animal owners, veterinarians, and farmers [145,146]. CPXV, a member of the Poxviridae family and the Orthopoxvirus genus, is known for causing a self-limiting, localized infection in humans, although more severe and occasionally fatal cases have been documented [147,148]. The virus is primarily transmitted to humans through direct contact with infected animals. Recent studies have highlighted the growing role of pet rats and free-roaming cats as vectors of the disease [148].

The cowpox virus (CPXV) belongs to the Orthopoxvirus species, and the disease it causes shares symptoms with smallpox. Historically, cowpox infections in humans were associated with direct contact with infected cows. However, in contemporary settings, other animals, particularly rodents, have emerged as significant reservoirs. Voles (Microtus arvalis and Myodes glareolus), field mice, and even domestic animals like rats and cats are now considered essential vectors of CPXV [149]. In 2009, an unusual outbreak in Munich was traced to infected pet rats, which had been purchased from a large breeder and subsequently spread the infection to human households [150,151]. The ability of infected rats to transmit CPXV, despite appearing asymptomatic, underscores the importance of recognizing potential reservoirs in domestic environments.

In humans, CPXV typically causes mild, localized lesions, often beginning as papules that progress to vesicles and pustules. After some time, these lesions form crusts. The incubation period for CPXV infection typically ranges from 8 to 12 days. While the majority of cases are self-limiting, severe cases with systemic involvement can occur, leading to complications such as lymphadenopathy (swollen lymph nodes) and, in rare instances, life-threatening conditions. In most cases, human infections occur through skin lesions following direct contact with infectious tissue or secretions from cats or infected rats [147,152,153,154]. Skin lesions, typically seen on the hands, face, and other exposed areas, can sometimes progress into ulcerations [155]. At the site of infection, papules develop within 7–12 days, progressing into vesicles and subsequently into painful hemorrhagic pustules and black crusts, often appearing on the hands, shoulders, or face. Infected individuals experience flu-like symptoms, nausea, and muscle pain. While the lesions are usually localized, severe cases requiring hospitalization have been reported [156]. Notably, infections in immunocompromised individuals may present with more severe systemic symptoms, resembling the severe course of infections caused by other orthopoxviruses like smallpox [134].

The diagnosis of CPXV infection in humans and animals is facilitated by molecular techniques, mainly quantitative PCR (qPCR). Suitable sample materials for diagnosis include crusts from skin lesions or swab samples of the lesions, and in cases involving animal carcasses, nasal epithelium or nasal septum samples may be used. However, CPXV infections are often diagnosed only after the disease has progressed to more advanced stages, as the initial skin lesions may be misdiagnosed as other, more common dermatological conditions [155].

Due to the zoonotic nature of CPXV, prevention strategies primarily focus on avoiding direct contact with infected animals, particularly rodents, which are significant reservoirs of the virus. The role of voles as primary reservoirs necessitates the need for effective control measures to minimize the spread of CPXV in environments where pet rats or free-roaming cats are present [149]. Public health guidelines emphasize the monitoring of animal populations and the proper handling of potentially infected animals to prevent transmission. Taken together, CPXV remains a rare but significant zoonotic pathogen with the potential to cause severe infections, especially in immunocompromised individuals. While it is primarily transmitted through contact with infected animals, particularly rodents, the rising incidence in domestic animals, such as cats and rats, highlights the importance of vigilance in both animal and human health monitoring. With ongoing environmental and demographic changes, further research into epidemiology, transmission dynamics, and control measures for CPXV remains essential to mitigate future outbreaks.

## 7. Coronaviruses

Since the identification of infectious bronchitis virus (IBV) in 1937 [157], coronaviruses have been found in numerous animal species and humans. Coronaviruses belong to the family *Coronaviridae*, order *Nidovirales*, and are classified into two subfamilies: *Orthocoronavirinae* and *Letovirinae*. *Orthocoronavirinae* includes four genera: *Alphacoronavirus (α-CoV)*, *Betacoronavirus (β-CoV)*, *Gammacoronavirus (γ-CoV)*, and *Deltacoronavirus (δ-CoV)*. Currently, 17 species of *α-CoV*, 12 of *β-CoV*, 2 of *γ-CoV*, and 7 of *δ-CoV* have been classified by the International Committee on Taxonomy of Viruses [158]. Coronaviruses are characterized by their diverse hosts, including birds, pigs, dogs, cats, cattle, and humans. All *γ-CoVs* in birds are classified as avian coronaviruses (ACoVs) regardless of host species, antigenicity, or genome identity [159,160].

Several *Alphacoronavirus* species have also been detected in rodents, such as the Luchang virus and Longquan Aa coronavirus [161]. These viruses have been identified in different rodent species, including rats and mice, in both rural and urban settings. Rodents have historically played a role in the emergence of human coronaviruses, with two known human coronaviruses (OC43 and HKU1) originating from rodent ancestors [162]. For instance, a study in China detected novel rodent *Betacoronaviruses* in regions affected by SARS-CoV and MERS-CoV outbreaks, suggesting a potential link between rodent viral reservoirs and human infections. Moreover, genetic sequencing of these viruses has revealed similarities to human-infecting strains, emphasizing their relevance in coronavirus epidemiology [163]. To date, all known rat coronaviruses belong to the genus *Betacoronavirus*, which comprises five subgenera: *Embecovirus*, *Hibecovirus*, *Merbecovirus*, *Nobecovirus*, and *Sarbecovirus*. Members of the subgenus *Embecovirus* include SDAV, PRCV, RCV-BCMM, RCV-W, RCV-NJ, RCV-CARS, and the novel *China Rattus Coronavirus* (ChRCoV) HKU24 [121]. Rodent-associated *Betacoronaviruses* are of particular concern due to their genetic similarities with known human coronaviruses. Some of these viruses share close phylogenetic relationships with *Severe Acute Respiratory Syndrome Coronavirus* (SARS-CoV) and *Middle East Respiratory Syndrome Coronavirus* (MERS-CoV), raising concerns about their zoonotic potential. Studies have identified *Betacoronavirus* strains in wild rodents, including *Murine Coronavirus* (MHV), which causes respiratory and neurological diseases in mice. This connection has raised concerns regarding their potential involvement in the transmission and persistence of other coronaviruses. The outbreak of SARS-CoV-2 was first recorded in Wuhan, China, in late 2019 and rapidly evolved into a global pandemic [164,165]. Initially, when vaccines and treatments were unavailable, the use of animal models was crucial for studying disease mechanisms and developing countermeasures. Among the early species examined for susceptibility to SARS-CoV-2 were Norway rats (Rattus norvegicus), house mice (*M. musculus*), and golden hamsters (Mesocricetus auratus) [166]. While initial studies suggested that wild-type SARS-CoV-2 did not efficiently infect house mice [167], emerging variants of concern (VOCs), such as beta (B.1.351) and gamma (P. 1), have demonstrated the ability to infect this species [168].

Additionally, several members of the *Cricetidae* family, including hamsters, have shown susceptibility to SARS-CoV-2, although disease severity varies among species [43,169]. Notably, the transmission of the virus from pet hamsters to humans has been documented, marking the first reported cases of zooanthroponotic transmission [170].

Beyond domesticated rodents, multiple wild species have been experimentally infected with SARS-CoV-2, including the bushy-tailed woodrat (*Neotoma cinerea* Ord, 1815), North American deer mouse (*Peromyscus maniculatus* Wagner, 1845), white-footed mouse (*P. leucopus* Rafinesque, 1818), and Eurasian bank vole (*Myodes glareolus* Schreber, 1780) [171,172,173]. While infection dynamics differ among species and virus strains, infected rodents can shed the virus for several days, and transmission to uninfected individuals through direct contact has been observed [167,173]. These findings raise concerns about the potential for SARS-CoV-2 to establish persistent reservoirs within rodent populations, which could complicate efforts to eradicate the virus.

A serological survey conducted in Germany between 2020 and 2022 examined 694 bank voles, 2 common voles (*Microtus arvalis* Pallas, 1778), 27 house mice, 97 Norway rats, and 8 Apodemus species. With the exception of one inconclusive sample from a common vole collected in 2021, all samples tested negative using a multispecies ELISA targeting the receptor-binding domain (RBD) of SARS-CoV-2 [174]. Although these findings suggest a low prevalence of SARS-CoV-2 in wild rodent populations, the demonstrated susceptibility of certain species, particularly to VOCs, highlights the need for continued monitoring. If SARS-CoV-2 were to become established in rodent populations, it could lead to new transmission cycles, complicating control and mitigation efforts.

Future research should prioritize large-scale serological and virological screening of rodents across different ecological settings to assess the potential long-term risks of rodent-mediated SARS-CoV-2 transmission. Given their historical role as viral reservoirs and their susceptibility to specific SARS-CoV-2 variants, rodents remain a key focus for surveillance. Although current evidence suggests a limited presence of the virus in wild populations, ongoing monitoring is crucial to prevent potential zoonotic spillovers.

## 8. The Role of Climate Change and Human Activity in the Emergence of Rodent-Borne Diseases

**Figure 5:**
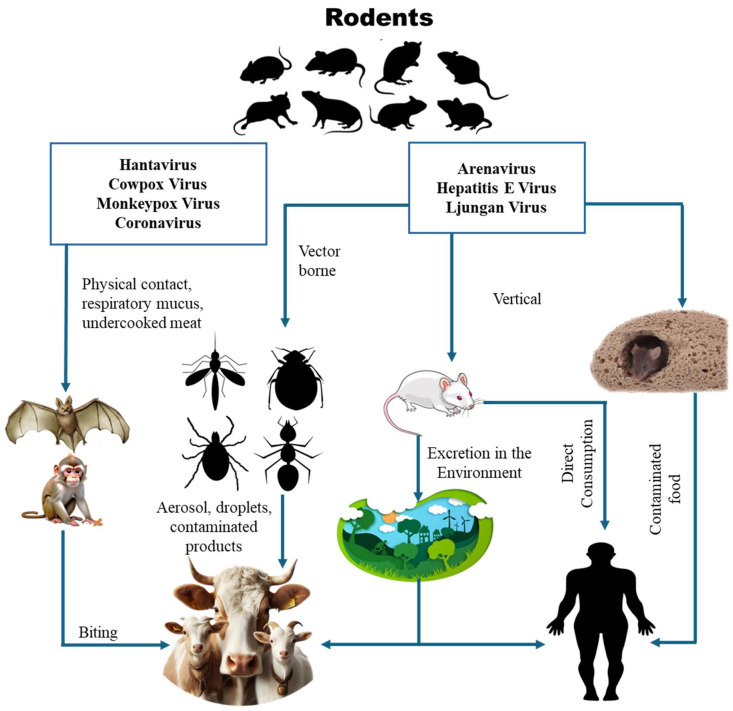
Transmission model of rodent-borne zoonotic viruses showing multiple routes of transmission.

These environmental shifts facilitate virus emergence and circulation, highlighting the urgent need for ecological monitoring and disease control [175,176,177]. Rodents significantly contribute to the spread of various diseases through both direct contact and indirect methods (Figure 5). Notably, these viruses can be transmitted into the environment through vertical transmission among rodents, direct consumption of rodents, or through food contaminated by rodents in certain countries.

(i) Warmer climates can expand the geographical range of rodent species, enabling them to thrive in new environments and increasing the risk of pathogen spillovers. Changes in seasonal rainfall can impact food availability, driving rodents into urban and peri-urban areas where contact with humans becomes more frequent, facilitating disease transmission [4,178,179]. Indeed, zoonotic transmission can arise in both livestock and human populations. Climate change significantly alters rodent habitats and behaviors, raising the likelihood of zoonotic disease spread. Rising temperatures and shifting precipitation patterns modify ecosystems, forcing rodents to adapt by migrating to new areas, including human settlements.
(ii) Climate changes alter pathogen dynamics by influencing the survival, replication, and transmission of pathogens transmitted by rodents. Rising temperatures and humidity levels can enhance the persistence of bacterial, viral, and parasitic pathogens in the environment, increasing their transmission potential. Additionally, altered rodent immunity and stress from environmental changes can lead to higher pathogen-shedding rates. These shifts contribute to the emergence and re-emergence of rodent-borne zoonoses, necessitating improved monitoring and adaptive public health interventions [180,181,182].
(iii) Extreme weather events, such as hurricanes, floods, droughts, and wildfires, have a significant influence on the emergence of rodent-borne zoonotic diseases [183,184,185]. Flooding can displace rodent populations, forcing them into human-inhabited areas, thereby increasing direct contact and contamination of water and food sources with pathogens, such as *Leptospira* spp. Droughts reduce natural food availability, driving rodents to forage in urban environments and heightening the potential for disease spillover. Wildfires destroy habitats, prompting rodent migration and altering predator–prey dynamics, which can potentially increase rodent densities [186]. These disturbances exacerbate the dissemination of pathogens, underscoring the need for proactive disease surveillance and environmental management in disaster-prone regions [187,188,189]. Changes in food availability due to altered vegetation cycles can lead to population surges, intensifying competition, and aggressive behaviors that enhance pathogen spread.
(iv) Anthropogenic activities, such as deforestation, agricultural expansion, and urbanization, exacerbate the risks associated with rodent-borne zoonoses. Habitat destruction forces rodents to migrate, often bringing them closer to human settlements, as well as animals. Climate-induced shifts in vector populations, including insects, further compound this risk by altering transmission cycles [13]. To mitigate the negative impacts of climate change and anthropogenic activities on the emergence of rodent-borne zoonotic diseases, it is crucial to implement coordinated efforts that include surveillance, environmental management, and control of rodent populations [180,190,191].

## 9. Conclusions and Recommendations

Rodents play a crucial role in transmitting many zoonotic diseases due to their adaptability, rapid reproduction, and close proximity to human environments. The increasing number of identified rodent-associated viruses underscores their significance as reservoirs for emerging infectious diseases, a threat that worsens with urbanization, environmental degradation, and climate change. A deeper understanding of rodent–virus interactions will be vital for improving global public health preparedness.

To address these risks, a comprehensive and proactive approach is necessary, one that integrates ecological understanding with public health strategies. The key recommendations include:

(i) Addressing the root causes of disease emergence: this involves multidisciplinary efforts, including surveillance, rodent control, and community education.
(ii) Enhancing surveillance and early warning systems: rodents are key reservoirs for many emerging viruses; therefore, integrating rodent population monitoring with molecular diagnostics and ecological monitoring, alongside climate and land use information, will allow for timely interventions.
(iii) Adapting to and mitigating climate change: Climate shifts influence rodent habitats, breeding cycles, and migration patterns, often expanding their range and increasing human exposure. Efforts should focus on reducing greenhouse gas emissions, conserving ecosystems, and promoting sustainable agriculture to minimize habitat disruption and help naturally regulate rodent populations.
(iv) Promoting community education campaigns: These campaigns should emphasize hygiene and food safety while highlighting the role of rodents in disease transmission and the importance of proper food storage and waste management to ensure long-term disease prevention. Finally, collaboration among governments, scientists, and public health agencies is essential for developing and implementing evidence-based policies to combat the increasing threat of rodent-borne zoonoses effectively.

## Figures and Tables

**Table 1:** Diseases caused by different hantaviruses, modified after Avšič-Županc et al. [24].

Disease	Virus	Reservoir Species
**Hemorrhagic Fever with Renal Syndrome**	Amur/Soochong	*Apodemus peninsulae*
	Dobrava	*Apodemus flavicollis*
	Hantaan	*Apodemus agrarius*
	Puumala	*Myodes glareolus*
	Saaremaa	*Apodemus agrarius*
	Seoul	*Rattus norvegicus* and *Rattus rattus*
	Tula	*Microtus arvalis*
**Hantavirus Cardiopulmonary Syndrome**	Anajatuba	*Oligoryzomys mattogrossae*
	Araucaria	*Oligoryzomys nigripes*, *Oxymycterus judex*, *Akodon montensis*
	Araraquara	*Necromys lasiurus*
	Bayou	*Oryzomys palustris*
	Bermejo	*Oligoryzomis chacoensis*
	Black Creek Canal	*Sigmodon hispidus*
	Castelo dos sonhos	*Oligoryzomys utiaritensis*
	Choclo	*Oligoryzomys fulvescens*
	Itapua	*Oligoryzomys nigripes*
	Juquitiba	*Oligoryzomys nigripes*
	Laguna Negra	*Calomys laucha; Calomys callidus; Calomys callosus*
	Lechiguanas	*Oligoryzomys flavescens*
	Maporal	*Oligoryzomys delicatus*
	Monongahela	*Peromyscus maniculatus*
	Neembucu	*Oligoryzomys chacoensis*
	New York	*Peromyscus leucopus*
	Oran	*Oligoryzomys longicaudatus*
	Paranoa	*Necromys lasiurus*
	Rio Mamore	*Oligoryzomys microtis*
	Sin Nombre	*Peromyscus maniculatus*
