# Correction: Shehata et al. The Hidden Threat: Rodent-Borne Viruses and Their Impact on Public Health. *Viruses* 2025, *17*, 809

**DOI:** 10.3390/v17101396

**Published:** 2025-10-21

**Authors:** Awad A. Shehata, Rokshana Parvin, Shadia Tasnim, Phelipe Magalhães Duarte, Alfonso J. Rodriguez-Morales, Shereen Basiouni

**Affiliations:** 1 TUM School of Natural Sciences, Bavarian NMR Center (BNMRZ), Structural Membrane Biochemistry, Technical University of Munich, 85748 Garching, Germany; 2 Department of Pathology, Faculty of Veterinary Science, Bangladesh Agricultural University, Mymensingh 2200, Bangladesh; rokshana.parvin@bau.edu.bd (R.P.); sadiatasnim562@gmail.com (S.T.); 3 Postgraduate Program in Animal Bioscience, Federal Rural University of Pernambuco (UFRPE), Recife, Pernambuco 52171-900, Brazil; duarte.phe@gmail.com; 4 Faculty of Health Sciences, Universidad Científica del Sur, Lima 15307, Peru; arodriguezmo@cientifica.edu.pe; 5 Grupo de Investigación Biomedicina, Faculty of Medicine, Fundación Universitaria Autónoma de las Américas-Institución Universitaria Visión de las Américas, Pereira 660003, Colombia; 6 Institute of Molecular Physiology, Johannes Gutenberg University, 55128 Mainz, Germany; sbasioun@uni-mainz.de

## Error in Table

In the original publication [1], some errors were identified in Table 1. The etiological agents of Hemorrhagic Fever with Renal Syndrome and Hantavirus Cardiopulmonary Syndrome were interchanged. Additionally, there were some inconsistencies in some of the reservoirs and Luxi virus was removed for not being associated with human infection yet. Finally, the reference cited as [18] in the table should be updated to [24]. The corrected Table 1 is presented below.

The authors affirm that the scientific conclusions remain unaffected, as the text in the preceding paragraph is accurate. These corrections have been approved by the Academic Editor, and the original publication has been updated accordingly.

## Figures and Tables

**Table 1:** Diseases caused by different hantaviruses, modified after Avšič-Županc et al. [24].

Disease	Virus	Reservoir Species
**Hemorrhagic Fever with Renal Syndrome**	Amur/Soochong	*Apodemus peninsulae*
	Dobrava	*Apodemus flavicollis*
	Hantaan	*Apodemus agrarius*
	Puumala	*Myodes glareolus*
	Saaremaa	*Apodemus agrarius*
	Seoul	*Rattus norvegicus* and *Rattus rattus*
	Tula	*Microtus arvalis*
**Hantavirus Cardiopulmonary Syndrome**	Anajatuba	*Oligoryzomys mattogrossae*
	Araucaria	*Oligoryzomys nigripes*, *Oxymycterus judex*, *Akodon montensis*
	Araraquara	*Necromys lasiurus*
	Bayou	*Oryzomys palustris*
	Bermejo	*Oligoryzomis chacoensis*
	Black Creek Canal	*Sigmodon hispidus*
	Castelo dos sonhos	*Oligoryzomys utiaritensis*
	Choclo	*Oligoryzomys fulvescens*
	Itapua	*Oligoryzomys nigripes*
	Juquitiba	*Oligoryzomys nigripes*
	Laguna Negra	*Calomys laucha; Calomys callidus; Calomys callosus*
	Lechiguanas	*Oligoryzomys flavescens*
	Maporal	*Oligoryzomys delicatus*
	Monongahela	*Peromyscus maniculatus*
	Neembucu	*Oligoryzomys chacoensis*
	New York	*Peromyscus leucopus*
	Oran	*Oligoryzomys longicaudatus*
	Paranoa	*Necromys lasiurus*
	Rio Mamore	*Oligoryzomys microtis*
	Sin Nombre	*Peromyscus maniculatus*

## References

[1] Shehata A.A., Parvin R., Tasnim S., Duarte P.M., Rodriguez-Morales A.J., Basiouni S. (2025). The Hidden Threat: Rodent-Borne Viruses and Their Impact on Public Health. Viruses.

